# Partial Injection Underwater Endoscopic Mucosal Resection for a Colorectal Flat Lesion

**DOI:** 10.1111/den.70095

**Published:** 2026-01-11

**Authors:** Hidenori Kimura, Kazuo Shiotsuki, Takuji Iwashita

**Affiliations:** ^1^ Division of Digestive Endoscopy, Department of Medicine Shiga University of Medical Science Otsu Shiga Japan; ^2^ Department of Gastroenterology Kanagawa Cancer Center Yokohama Kanagawa Japan; ^3^ Division of Gastroenterology, Department of Medicine Shiga University of Medical Science Otsu Shiga Japan

**Keywords:** colorectal neoplasms, partial injection, underwater endoscopic mucosal resection

## Abstract

Watch a video of this article.

Colorectal underwater endoscopic mucosal resection (UEMR) is widely performed because of its higher en bloc and R0 resection rates, as well as lower local recurrence rates compared with conventional EMR [1, 2]. However, identifying the oral side of a lesion can occasionally be challenging in underwater conditions, leading to piecemeal resection. Here, we demonstrate a technique for partial submucosal injection on the oral side during UEMR (PI‐UEMR) for a flat colorectal lesion. A 60‐year‐old man underwent colonoscopy, which revealed a 13‐mm flat reddish lesion in the transverse colon (Figure 1a). Endoscopy with narrow‐band imaging showed an irregular surface and vessel pattern, suggesting an advanced adenoma (Figure 1b). Underwater conditions made it difficult to continuously visualise the oral side of the lesion without the assistance of a sheath, raising concerns regarding the possibility of piecemeal resection (Figure 1c). Therefore, we decided to perform PI‐UEMR. After a partial submucosal injection of 3 mL of saline solution on the oral side of the lesion, the overall visualisation improved (Figure 1d). We captured the lesion while maintaining the snare tip on the oral side. En bloc resection was achieved without any complications (Figure 1e, Video S1). Pathological examination revealed a high‐grade adenoma with tumour‐free margins (Figure 2). PI‐UEMR, which involves local injection only on the oral side of the lesion, can improve the visibility of the oral margin while maintaining the floating effect [3], an original advantage of the underwater resection technique. A previous report demonstrated that PI‐UEMR achieved better treatment outcomes than conventional UEMR in the duodenum [4]. The detailed presentation of this case not only suggests the potential applicability of PI‐UEMR to colorectal flat lesions for which piecemeal resection is a concern with conventional UEMR [5], but also may contribute to the adoption of this technique as a simple and reproducible procedure.

**FIGURE 1:**
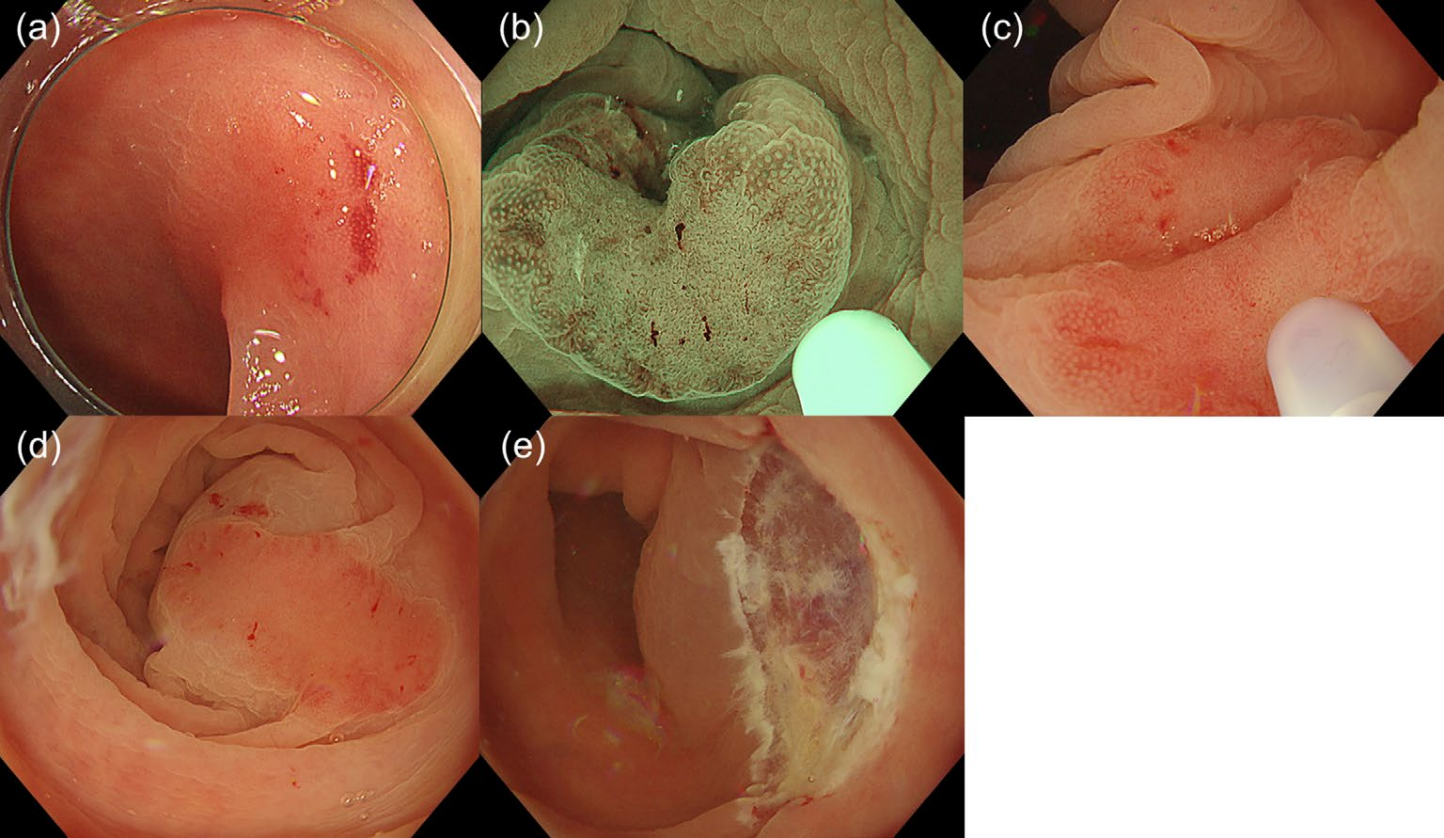
Partial injection underwater endoscopic mucosal resection (PI‐UEMR) procedure. (a) Colonoscopy shows a 13‐mm reddish flat lesion, located in the transverse colon. (b) Endoscopy with narrow‐band imaging showed an irregular surface and vessel pattern, suggesting an advanced adenoma. (c) Underwater conditions made it difficult to continuously visualise the oral side of the lesion without the assistance of a sheath. (d) After partial submucosal injection using saline solution on the oral side of the lesion, overall visualisation improved. (e) En bloc resection was achieved.

**FIGURE 2:**
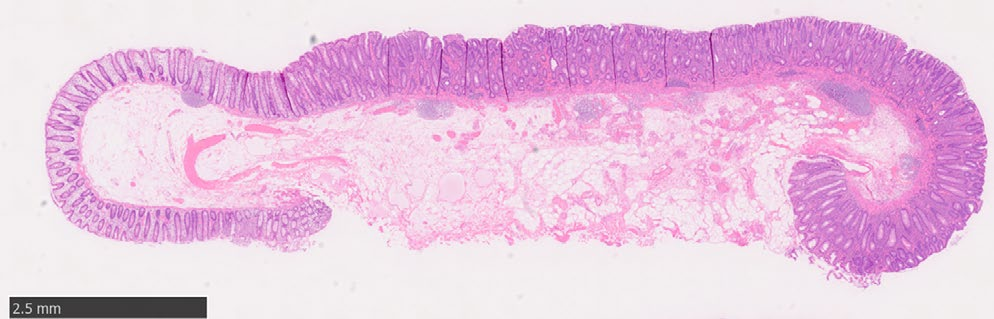
Pathological examination revealing a high‐grade adenoma with tumour‐free margins.

## Author Contributions

H.K.: conception and design of the study. H.K., K.S. and T.I.: drafting and revision of the manuscript and final approval of the manuscript.

## Funding

The authors have nothing to report.

## Ethics Statement

The authors have nothing to report.

## Conflicts of Interest

The authors declare no conflicts of interest.

## Supporting information


**Video S1:** Colonoscopy revealed a flat, reddish lesion in the transverse colon. The lesion was successfully removed using partial injection underwater endoscopic mucosal resection.
